# REACTIONS TO PERPETRATORS OF BENEVOLENT AGEISM: WHAT INFLUENCES PERCEPTIONS?

**DOI:** 10.1093/geroni/igad104.0201

**Published:** 2023-12-21

**Authors:** Alison Chasteen, Zainab Saleem, Michelle Horhota

**Affiliations:** University of Toronto, Toronto, Ontario, Canada; University of Toronto, Toronto, Ontario, Canada; Furman University, Greenville, South Carolina, United States

## Abstract

Ageism against older adults is a longstanding and pervasive problem. Benevolent ageism can be particularly difficult to address, given it is characterized by warmth and condescension. In two studies, we examined the impact of confronting prejudice on perceptions of a young adult perpetrator who expressed benevolent ageism. In both studies, the perpetrator initiated unwanted help to an older person who was either 62 or 82 years old, and the older adult either accepted or politely rejected the unwanted assistance. In both studies, participants felt that rejecting (i.e., confronting) the ageist behavior decreased the likelihood the perpetrator would repeat the behavior. Young adult participants used the age of the older target as a cue for interpreting the perpetrator’s intentions, such that they rated the perpetrator’s actions as less intending to offend if the older target was 82 than 62 years of age. Moreover, younger adults used the age of the older target to determine whether the perpetrator should be confronted, indicating that confrontation should occur when the target was 62 vs. 82 years old. Taken together, these studies demonstrate that participants of all ages feel that confronting benevolent ageism can be an effective tool for reducing biased behavior. As well, these findings show that the age of the older adult target might be used as a normative cue to interpret the perpetrator’s intentions and actions, thus providing a more nuanced picture of what might drive witnesses’ responses to ageist behavior.

